# AD04 – modifying Alzheimer’s Disease by modulation of neuroinflammation

**DOI:** 10.1002/alz.095657

**Published:** 2025-01-09

**Authors:** Carmela R Abraham, Achim Schneeberger, Agustin F Santana III, Thomas Hoffmann, Anita Fischer, Sylvie Trembleau

**Affiliations:** ^1^ Advantage Therapeutics, Inc., Miami, FL USA

## Abstract

**Background:**

We aim to repurpose the widely used adjuvant and immunomodulator Alhydrogel^TM^ at a specific dose and schedule (AD04) as a disease‐modifying treatment for mild Alzheimer’s disease (AD). AD04 is a potent adjuvant commonly used in vaccines and allergy immunotherapies to augment the magnitude and durability of the immune response. Our preliminary data come from a clinical Phase 2 study in which AD04 served as a control arm. In this study, AD04 showed promising results in delaying hippocampal atrophy and cognitive decline, as well as improving the quality of life in patients with mild AD. A global statistical test (GST) was used to compare the treatment effect of AD04 to other drugs/drug candidates. GST used is a combination of ADAS‐cog, ADCS‐ADL and the CDR‐sb. As such, it combines three important perspectives of three different aspects of AD progression. The effect size seen with AD04 is comparable or better than that of most other available treatments. However, the precise mode of action of subcutaneous (s.c.) administration of AD04 on the brain has not been deciphered.

**Method:**

Aged (24 mo) C57BL/6 mice were injected s.c. with AD04 or with PBS, three times, and 3‐5 days after the last injection they were tested for fear conditioning. One day later, the mice were sacrificed, and hippocampi and peripheral organs were subjected to proteomics. Young (10wks) mice were used as controls.

**Result:**

In the fear conditioning test, AD04‐treated old mice, exhibited an improved freezing behavior compared with the PBS‐injected mice. Proteomics analysis of the hippocampi revealed that peripheral AD04 administration modulated the production of proteins involved in lipid metabolism regulation and affected microglial phenotype into a phagocytic type.

**Conclusion:**

Based on these preclinical results we conclude that AD04 administration induces microglia to phagocytose brain debris, such as apoptotic neurons, synapses and degraded myelin in the aged brain, and in the AD brain also Abeta, extracellular tau tangles and oxidized lipids. By changing the microglial phenotype, AD04, indirectly reduces the neuroinflammation induced by Abeta, tau and neuronal debris, leading to an anti‐inflammatory milieu in the brain which protects and rescues the hippocampus from neurodegeneration.

